# Basic Substances and Nanotechnology: Bridging Sustainability and Innovation for Fungal Disease Management in Plants

**DOI:** 10.3390/plants15050689

**Published:** 2026-02-25

**Authors:** Antigoni Akrivou, Nikolaos Tsiropoulos, Evangelos Karanasios, Emilia Markellou, Panagiotis Madesis

**Affiliations:** 1 Department of Agriculture Crop Production and Rural Environment, University of Thessaly, 38446 Volos, Greece; antakrivou@uth.gr (A.A.); ntsirop@uth.gr (N.T.); 2 Scientific Directorate of Pesticides’ Control & Phytopharmacy, Benaki Phytopathological Institute, 14561 Kifissia, Greece; e.karanasios@bpi.gr; 3 Scientific Directorate of Phytopathology, Benaki Phytopathological Institute, 14561 Kifissia, Greece

**Keywords:** chitosan, nanoparticles, natural compounds, plant disease management, plant fungi, sustainability, Regulation EU 1107/2009

## Abstract

Plant diseases caused by fungi remain a major challenge for global agriculture, undermining both food security and ecosystem resilience. The increasing concern about the use of chemical pesticides, the onset of resistance, and environmental pollution are accelerating the search for effective and more sustainable plant protection alternatives. In this context, basic substances have emerged as prominent solutions for integrated pest management because they are naturally derived and have consistently been shown to present a low risk to human health and the environment. In the meantime, advances in green nanotechnology are enhancing their functional potential through improved stability, bioavailability, and targeted delivery. By reducing application rates and decreasing residues, environmentally friendly nanoformulations, which may be produced using biopolymers, plant extracts, or mineral precursors, would have greater durability. These innovations are in line with the Farm to Fork Strategy under the European Green Deal and the United Nations’ 2030 Agenda for Sustainable Development, enabling the transition to more sustainable food systems. Addressing challenges related to safety evaluations of nanoformulations and production scalability will contribute to public acceptance. This review synthesizes current scientific advances in the formulation and application of natural compound-based nanoformulations alongside evolving policy frameworks and identifies pathways to integrate harmonized risk-assessment approaches into future governance. It aims to promote the adoption of nano-based natural substances as key elements of next-generation sustainable crop protection strategies.

## 1. Introduction: The Need for New Tools in Plant Fungal Disease Management

Plant pathogens are major causes of crop diseases and threaten agriculture worldwide, causing significant yield and quality losses [1,2]. The increased mobility of humans and international trade in agricultural commodities have led to the rapid dispersal of pests and pathogens into new geographic regions [3,4]. The distribution, virulence, and life cycles of pests and diseases are impacted by changes in temperature and precipitation patterns caused by climate change [5,6,7]. At the same time, these environmental changes can also increase host vulnerability and disrupt interactions with natural enemies and vectors [8]. The coexistence of multiple pathogens in crops requires broad-spectrum, multi-functional management strategies [9]. Synthetic fungicides have long constituted the principal strategy for fungal disease management, but their overuse has been associated with serious environmental, health, and resistance problems [10]. Long-term fungicide applications can deplete soil microbiomes, which erode the resilience of agroecosystems [11], while climate change may further compromise the efficacy of fungicides [12]. In this context, a 50% reduction in pesticide use and risk by 2030 has been mandated by the Farm to Fork Strategy of the European Union (EU), along with 25% of the agricultural land under organic farming [13,14]. By 2023, a 58% decrease in pesticide use and risk was already observed compared to the 2015-2017 baseline [15]. Attainment of these sustainability goals calls for a switch towards safer, low-risk alternatives to conventional pesticides [16].

Basic substances, including naturally occurring compounds, minerals, plant extracts, and biochemicals, have emerged as promising eco-friendly options for controlling fungal diseases in plants. Regulation (EC) No. 1107/2009 [17] defines verbatim basic substances as certain substances which are not predominantly used as Plant Protection Products (PPP), but which may be of value for plant protection; however, the economic interest of applying for approval may be limited. Therefore, specific provisions should ensure that such substances, as far as their risks are acceptable, may also be approved for plant protection use. In particular, Article 23 of Regulation (EC) No. 1107/2009 lays down specific criteria to identify an active substance as eligible as basic: (a) is not a substance of concern as defined in Article 3(4) of the PPP Regulation; (b) does not have an inherent capacity to cause endocrine-disrupting, neurotoxic or immunotoxic effects; (c) is not predominantly used for plant protection purposes but nevertheless is useful in plant protection either directly or in a product consisting of the substance and a simple diluent; and (d) is not placed on the market as a plant protection product. As of February 2025, a total of 28 basic substances have been approved for use in plant protection within the EU. Two (1,3,7-trimethylxanthine (caffeine) and eggshell powder) have been rejected, and two (*Quassia amara* L. wood extract and sodium hypochlorite) are pending approval. A total of 27 out of 28 approved basic substances can be used against plant pathogens (except for *Onobrychis viciifolia* (sainfoin) dried pellets), and 20 of these have demonstrated fungicidal activity, particularly for the protection of fruits and vegetables [18].

Nanotechnology has become a revolutionary and complementary approach to sustainable agriculture throughout this shift [19]. A nanoparticle is defined verbatim by the European Union [20] as a natural, incidental or manufactured material consisting of solid particles that are present, either on their own or as identifiable constituent particles in aggregates or agglomerates, and where 50% or more of these particles in the number-based size distribution fulfill at least one of the following conditions: (a) one or more external dimensions of the particle are in the size range from 1 nm to 100 nm; (b) the particle has an elongated shape, such as a rod, fiber or tube, where two external dimensions are smaller than 1 nm and the other dimension is larger than 100 nm; and (c) the particle has a plate-like shape, where one external dimension is smaller than 1 nm and the other dimensions are larger than 100 nm. In the determination of the particle number-based size distribution, particles with at least two orthogonal external dimensions larger than 100 μm need not be considered. However, a material with a specific surface area by volume of <6 m^2^/cm^3^ shall not be considered a nanomaterial.

Nanotechnology enables the engineering of advanced materials and delivery systems with novel physicochemical properties [21]. In the EU regulatory framework, nanomaterials fall under the existing provisions of Registration, Evaluation, Authorisation and Restriction of Chemicals (REACH) and Classification, Labeling and Packaging of Substances and Mixtures (CLP), thereby ensuring that their use in agriculture is subject to appropriate safety and classification mechanisms [22]. What distinguishes nanomaterials is their high surface area and enhanced reactivity, enabling precise delivery, controlled release, and targeted interaction with plant and pathogen systems [23]. Consequently, nanotechnology can reduce input use, improve yields, and enhance resource efficiency [24]. Moreover, nanoparticles (NPs) can function both as protectants and as carriers, improving the performance of biocontrol agents and natural compounds. Integrating nanotechnology with basic substances thus represents a powerful step toward sustainable, efficient, and resilient plant protection systems [25]. The unique contributions and conceptual advances of this review are summarized in Box 1.

Box 1 **Novel and Conceptual Advances of this Review.**

**Regulatory-Technical Synthesis:** Directly bridges the gap between EU Regulation (EC) 1107/2009 for basic substances and nanotechnology.

**Mechanistic Benchmarking:** Establishes chitosan as a functional “model substance” to provide a blueprint for the nano-conversion of other basic substances.

**Transferability Analysis:** Identifies specific factors (environmental, biological and ecological complexity, operational and application precision) that explain the challenge of transferring laboratory success to the field.

**Pathosystem Evaluation:** Provides a structured analysis of diverse pathosystems, evaluating why specific modes of action succeed or fail in “real-world” environments.

**Hurdles and Gap Assessment:** Evaluates the toxicological and regulatory “blind spots”of nanoformulations, including batch-to-batch heterogeneity, occupational exposure for farmers, and the lack of nano-specific provisions in current EU frameworks.

**Innovation Roadmap:** Suggests a transition toward the Safe- and Sustainable-by-Design framework, integrating AI-based precision application and molecular breeding to enhance plant response to nano-delivered elicitors.

## 2. From Mode of Action to Agricultural Practice: Technological Achievements and Practical Potential

Nanoparticles are classified into carbon-based, inorganic, and organic types. Carbon-based NPs include materials derived from carbon structures and allotropes with high surface area and conductivity like graphene and its derivatives (including graphene oxide), carbon black which is utilized as a conductive filler in industrial applications, carbon quantum dots (<10 nm) which are valued for their biocompatibility and fluorescence, and fullerenes-C_60_ with characteristics that modulate reactive oxygen species (ROS). Inorganic NPs include magnetic ones like iron, nickel, cobalt and their oxides that react to magnetic fields; ceramics like alumina, silica, titanium dioxide, and hydroxyapatite that are highly stable and used in drug delivery and tissue engineering; and semiconductors like zirconium dioxide, and zinc oxide with intermediate conductivity that are appropriate for sensors, coatings, and biomedical applications. Organic NPs are generally biodegradable and biocompatible, composed of proteins, lipids, polymers, or carbohydrates. Their subtypes include polymeric NPs (such as chitosan, alginate, polylactic acid, and polyglycolic acid) for drug/gene delivery, lipid-based ones represented by liposomes, solid lipid NPs, and nanostructured lipid carriers which can all encapsulate a wide range of molecules. There are also carbohydrate-based NPs, represented by starch, and dextran used in biomedical, cosmetic, and food industries [26,27].

Basic substances combat fungal pathogens through several modes of actions. They may stimulate plant defense responses (chitosan from *Aspergillus niger*, chitosan hydrochloride, fructose, and sucrose), exhibit direct antifungal activity (*Allium cepa* L. bulb extract, calcium hydroxide, cow milk, *Equisetum arvense* L., hydrogen peroxide, lecithins, mustard seed powder, *Salix* spp. cortex, sodium chloride, sodium hydrogen carbonate, sunflower oil, talc E553B, *Urtica* spp., vinegar, *Vitis vinifera* seed extract, whey) or create protective physical barriers like clayed charcoal. The list of approved uses of basic substances for fungal disease management in plants and the pathosystems, under Regulation (EC) No. 1107/2009 [18] are presented in Table S1. The agricultural potential of basic substances herein is based on greenhouse and field trials only. While greenhouse studies demonstrate the maximum potential efficacy under controlled conditions, field trials reveal the environmental resilience of these treatments. The transition of basic substances from controlled environments to agricultural practice reveals a performance gap dictated by the interaction between the substance’s chemical nature and the pathogen’s infection patterns. An analysis of the approved basic substances (Table S1) and literature review reveals that their efficacy is not universal but is connected to their functional properties.

Inorganic compounds are substances such as calcium hydroxide, hydrogen peroxide, sodium chloride, and sodium hydrogen carbonate, and are predominantly approved for managing diseases caused by biotrophic pathogens including mildews, rusts, and scabs. These substances function through rapid osmotic and pH modulation; by creating an alkaline environment or high osmotic pressure on the leaf surface, they physically dehydrate fungal spores and inhibit germination. Talc E553B and clayed charcoal serve as effective physical barriers; by coating the leaf surface, they prevent foliar fungi from adhering to or penetrating host tissues. Soil-applied biochar at rates from 1% to 5% proved effective at inhibiting the pathogens *Leveillula taurica* (powdery mildew) and *Botrytis cinerea* (gray mold) in pepper and tomato plants. The efficacy was significant across both systems, with researchers recording up to a 65% reduction in gray mold severity on tomato leaves and a marked delay in powdery mildew development on pepper plants [28]. A study conducted by Rao and Tewari [29] demonstrated that calcium hydroxide is effective at suppressing American leaf spot of coffee, caused by the fungus *Mycena citricolor* in a dose-dependent manner. Spray application of 0.1 mg/cm^2^ calcium hydroxide resulted in a 100% inhibition of lesion development on coffee leaves by neutralizing the oxalic acid secreted by the pathogen and forming insoluble calcium oxalate crystals. Even at lower doses, such as 0.04 mg/cm^2^, the treatment significantly reduced infection rates to roughly 32-36%. In Egypt, field application of 1.2 g/L hydrogen peroxide in combination with the biocontrol agent *Trichoderma harzianum* against *Sclerotium rolfsii*, the causal agent of sunflower root and collar rot, achieved sunflower protection of 78% against *S. rolfsii* infection [30]. Furthermore, in greenhouse experiments, NaCl treatments significantly suppressed Fusarium crown and root rot caused by *Fusarium oxysporum* and *F. proliferatum*, respectively, while sodium hydrogen carbonate at 0.12 and 0.24 mol/L significantly reduced the disease severity of *Puccinia triticina* from 11.4% to 2.2% compared with the water-treated control [31,32].

Lipids and hydrophobic films: Lecithins and sunflower oil function by forming a hydrophobic film that prevents moisture accumulation, hindering the attachment of spores like *Alternaria cichorii* and *Plasmopara viticola* onto leaf surfaces. In greenhouse trials, 10% sunflower oil concentration demonstrated significant effectiveness by reducing late blight (*Phytophthora infestans*) symptoms on potato leaves compared to untreated controls, whereas a lower 1% concentration was found to be ineffective. In field trials, the application of the 10% emulsion provided effective yield protection by maintaining the photosynthetic area of the leaves and extending the vegetation period, which ultimately resulted in an increased tuber yield [33]. Soya lecithin at a concentration of 0.75 mL/L was applied five times throughout the growing season and tested against the fungal pathogen *Alternaria cichorii* on several varieties of endive (*Cichorium endivia* L.). The treatment resulted in a lower infection index representing roughly 5% to 10% infected leaf area compared to the significantly higher levels of infection observed in untreated plots [34].

Carbohydrates and dairy-based products like fructose, sucrose, whey and milk highlight the role of “sweet immunity”. They serve as signaling molecules that trigger defense mechanisms by upregulating peroxidases and PR proteins [35] which make them effective when applied preventively for the control of powdery mildews where the goal is to prime the plant before infection by the pathogens occurs. In field trials the efficacy of milk and whey powders was evaluated for the management of powdery mildew (*Erysiphe necator*) on grapevine (*Vitis vinifera* L.). The dairy products were applied via foliar spray at concentrations of 10, 20, and 30 g/L for milk powder and 30 and 45 g/L for whey powder. Results indicated that these basic substances provided a significant reduction in disease compared to the untreated control, achieving a performance comparable to a standard sulfur-based fungicide. Specifically, the 30 g/L milk powder rate demonstrated a disease severity reduction of 95-99% and numerically outperformed the sulfur reference in late-season bunch assessments. Similarly, the higher dosage of whey powder (45 g/L) showed improved disease control relative to lower concentrations [36].

Volatile compounds and organic acids include vinegar, *Allium cepa*, and mustard seed powder that act as bio-fumigants. Their volatile sulfur compounds and organic acids disrupt internal cellular processes which make them effective for seed treatments (e.g., *Tilletia caries*) and soil-borne wilt management in wheat, where inorganic compounds and salts fail. The seeds of the spring wheat cultivar “Laura” were treated at a rate of 2 g/kg or 4 g/kg of acetic acid vapor for one hour. In terms of efficacy, the 4 g/kg rate reduced infection of wheat plants by the pathogens *Tilletia tritici* and *T. laevis* by 81–86% the first year, while 100% control was achieved the second year, and was the same or similar to the effectiveness of a treatment with carboxin [37]. In a greenhouse, pot-based study, the authors evaluated the efficacy of tillered onion bulb extract for the control of melon wilt disease caused by the soil-borne pathogen *Fusarium oxysporum* f. sp. *niveum*. Studies of onion bulb extract demonstrated a clear dose–response effect: although the rate of 1000 mg/mL achieved the highest mycelial inhibition (73.15%), the rate of 250 mg/mL was selected for practical use. This adjusted rate provided >63% disease control in both resistant and susceptible varieties without compromising plant biomass. Mechanistically, the 250 mg/mL rate activated the plant’s defense mechanisms, with protective enzyme activity peaking at 72 h post-inoculation [38]. Similarly, mustard seed (*Brassica nigra*) extract at rates of 10 to 15 tons per hectare (soil application) significantly suppressed *Fusarium oxysporum* and *Rhizoctonia solani*, while simultaneously improving growth parameters like shoot length [39].

Plant defense inducers such as *Equisetum arvense*, *Salix* sp. cortex, and *Urtica* spp. trigger the plant’s internal immune system and enzyme production, showing a broader spectrum of activity including soil-borne pathogens (Fusarium, Sclerotium) and necrotrophs (*Botrytis cinerea*), as shown in Table S1. The efficacy of these substances stems from their complex secondary compounds, such as sulfur found in onion and mustard or the salicin of willow cortex. These act as natural fumigants in the soil or metabolic inhibitors on the leaf. Unlike salts, these substances can penetrate fungal membranes and disrupt internal cellular processes, which is why they are prioritized for soil-borne pathogens that salts cannot manage effectively. A study evaluated the use of horsetail extract against *Podosphaera pannosa* in cut roses at the rate of 4 g/L at weekly intervals across various cultivars. The infected leaf area reduced significantly without affecting plant growth [40]. The rate of 1% *Salix* cortex extract, when applied preventively for the control of grapevine downy mildew (*Plasmopara viticola*), reduced disease severity by ca. 70% [41]. In field trials conducted by Ghazal et al. [42], nettle extracts (*Urtica dioica*) were evaluated for their ability to manage gray mold in tomato plants. When applied as a control agent at a concentration of 30 mL/L, the methanolic extract (MU) reduced disease severity on leaves to 27% (infected leaf area), while the aqueous extract (QU) resulted in a severity of 29%. On average, across all concentrations tested in the greenhouse, the methanolic and aqueous nettle extracts maintained similar performance levels, with mean disease severity ratings ca. 34% compared to ca. 82% of the untreated plants. Beyond direct suppression, these extracts were also tested for their ability to induce resistance by spraying the plants four hours prior to pathogen inoculation. In these trials, the 30 mL/L concentration of MU reduced disease severity to 23%, while the aqueous version QU reduced to 24%. This defensive response is supported by biochemical data, which showed that the application of *U. dioica* extracts significantly increased the accumulation of total phenols in the tomato leaves.

Due to its versatile characteristics as a basic substance with a broad commercial use against biotrophic and necrotrophic fungi, chitosan is analyzed as a model basic substance in Section Chitosan: The Benchmark Model for Nano-Enabled Plant Protection.

In brief, integrating basic substances into field-level practices requires a systems-based approach that accounts for crop phenology, weather, and disease pressure. However, reproducibility is often hindered by the instability of organic molecules and a lack of standardized raw materials. Unlike persistent synthetic fungicides, substances like botanical extracts or dairy products are highly susceptible to UV degradation and variant rainfastness properties. For instance, a single rain event can remove water-soluble molecules like fructose or lecithin, reducing their efficacy from near-total inhibition of pathogens in controlled environments to negligible levels of inhibition in the field. Furthermore, the chemical profile of plant-based extracts fluctuates significantly based on the host plant’s origin, harvest timing, and extraction method, making it difficult to replicate a specific minimum effective rate across different studies. This challenge is further reinforced by the complexity of the field pathosystem, where the host–pathogen–environment interaction is far more complicated. In soil applications, for instance, the bio-fumigant properties of mustard seed or onion extracts are often neutralized by resident soil microbiota that utilize these organic compounds as carbon sources before they inhibit pathogens like *Sclerotium rolfsii*. Additionally, the physiological state of a field-grown plant under abiotic stress like drought or heat may prevent the successful activation of SAR typically triggered by elicitors like chitosan. Consequently, the transition to nano-enabled formulations is not just a technological upgrade but a necessary step to solve these transferability issues. By encapsulating these substances into lipid or polymeric nanocarriers, the active ingredients are shielded from UV degradation and premature microbial breakdown, providing a controlled release mechanism that maintains the “immune priming” effect over a longer duration, regardless of external environmental fluctuations.

### Chitosan: The Benchmark Model for Nano-Enabled Plant Protection

While the EU recognizes a diverse array of basic substances, chitosan is established here as the model basic substance due to its unique position at the intersection of regulatory approval and nanotechnological versatility. While inorganic compounds like calcium hydroxide rely on pH modulation and botanical extracts suffer from harvest-related variability, chitosan provides a stable biopolymeric scaffold but its effectiveness across different applications can be influenced by particle size, molecular weight, crystalline structure, level of deacetylation, and surface area [43]. Chitosan is a safe, nontoxic, biodegradable, and biocompatible polycationic polymer [44]. Its positively charged ions bind to negatively charged microbial surfaces, disrupting membrane integrity [45]. This interaction results in the leakage of vital cellular constituents, and, ultimately, the death of the targeted cells. Chitosan also causes the surrounding cells to become more resistant to pathogens, since it acts like a plant defense inducer [46]. It triggers innate immunity by stimulating the production of enzymes like chitinases [47], peroxidases and other markers of oxidative stress, leading to the accumulation of ROS that limit the growth of the pathogens [46,48]. Moreover, it results in the upregulation of key defense genes and the production of signaling molecules such as SA, jasmonic acid (JA), and ethylene through which SAR and induced systemic resistance (ISR) are activated [49,50]. Apart from mediating such complex immune reactions, chitosan has also been reported to enhance secondary metabolite production in plants, including phenolic compounds that reinforce the plant structural and chemical barriers [51].

Natural compound-based nanoformulations combine the intrinsic bioactivity of natural substances with the functional advantages conferred by nanotechnology. In recent years, advances in green chemistry have aligned with nanotechnology to create a new generation of low-risk, bioactive formulations. Green nanotechnology enables natural compounds to be transformed into nanoscale delivery systems with improved stability, solubility, bioavailability, and targeted delivery [52,53]. Reducing particle size and enhancing surface reactivity can increase adhesion to plant tissues, prolong the release of active ingredients, and intensify interactions with pathogens, thereby improving efficacy at lower application rates [54,55]. Furthermore, combining nanoformulations with phytohormones like SA enhances their performance by stabilizing the molecules, allowing for controlled release, and strengthening plant defense signals. This synergy provides benefits beyond those of individual components, leading to improved plant growth and stress resistance [56].

Recent progress in nanotechnology has further enhanced the antifungal efficacy of chitosan and other natural compounds, hence making it a very promising tool against a wide range of fungal pathogens. Due to their extremely small size and large surface area, chitosan nanoparticles (NCS) exhibit activity against fungi which is exerted by damaging the fungal cell walls and plasma membranes and thereby inducing cytoplasmic leakage [46]. Specifically, innovative green synthesis of NCS has shown significant inhibitory activity against *Botrytis cinerea* on strawberry leaves [57]. Furthermore, greenhouse trials demonstrated that the application of NCS prior to inoculation significantly suppressed Fusarium head blight. Compared to untreated controls, NCS-treated wheat showed a marked delay in pathogen colonization and disease progression. Four weeks post-inoculation, the disease severity in NCS-treated wheat plants was estimated at 41.77% [58].

In addition, loading NCS with bioactive compounds such as SA or JA has further augmented their effectiveness. Recent experiments (2024-2025) have elucidated the molecular crosstalk involved in these treatments, such as the manipulation of ER-protein quality control, to support host-induced defense against biotrophic fungi [59]. Notably, greenhouse studies have confirmed that NCS loaded with JA successfully primed plant resistance and suppressed *B. cinerea* infection by activating systemic defense pathways [60], thereby triggering robust defense responses.

A critical distinction must be made regarding the role of other basic substances in this nanotechnological framework. While chitosan is unique as a structural biopolymeric scaffold, other basic substances possess equal potential to be transformed into nanoscale delivery systems. Botanical extracts, such as *Salix* cortex or *Allium cepa* L. bulb extracts, often serve as active ingredients within lipid nanocarriers or act as reducing agents in the green synthesis of metallic nanoparticles. The aforementioned substances facilitate the particle’s physical synthesis but are not necessarily the “active ingredient” exerting the antifungal effect. Similarly, inorganic compounds like sodium hydrogen carbonate or calcium hydroxide may serve as precursors for mineral-based nanoparticles, while substances such as talc E553B or clayed charcoal can be downsized to the nanoscale to improve surface coverage.

## 3. Gaps and Hurdles in the Use of Natural-Compound Nanoparticles for Plant Protection

Even though natural-compound nanoparticles are environmentally friendly alternatives to conventional PPP, their application as effective plant-protection solutions is currently constrained by several regulatory, scientific, socioeconomic, and technical issues. A primary concern remains the gap between laboratory outcomes and field performance. Although strong antifungal or inducer activity is often demonstrated under laboratory conditions, performance under natural conditions tends to fluctuate; a variation that may arise from the disproportionately small number of field assessments compared to the extensive laboratory-based evidence [61,62]. A second challenge is the physical and chemical heterogeneity of the nanoparticles of natural compounds. This includes high batch-to-batch variability in terms of particle size, polydispersity, surface functionality, and encapsulation efficiency, all of which have a significant impact on biological performance and toxicological profiles. Such variability is widely reflected in green-synthesized natural-compound nanoparticles. Plant extracts and other natural substances introduce complex and variable phytochemical compositions that render reproducibility difficult [63]. This inconsistency complicates the elaboration of standardized doses, even more so when it comes to regulatory assessment. Significantly, it severely limits cross-study comparison. Furthermore, many natural-compound nanoparticle systems have very limited long-term stability. They are often aggregated or deteriorate due to ion strength, thermal fluctuations, or UV irradiation, typical in agricultural environments [64,65]. For dependable and scalable results, better formulation variable management is therefore essential.

Significantly, there are still gaps in our understanding regarding environmental persistence, behavior, and fate since the release dynamics, transport, and degradation of nanoformulations can differ from those of conventional pesticides. Natural-compound nanoparticles can experience changes like dissolution, surface oxidation, corona formation, and aggregation when exposed to soils, irrigation water, and plant surfaces. These “aging” processes can significantly alter mobility, bioavailability, and toxicity, yet are still not well characterized [64,66,67]. Recent environmental nanotoxicology studies [68] emphasize that nanoparticle transformations in response to pH, ion strength, salinity, organic matter, and redox conditions significantly affect behavior across environmental compartments. Similarly, knowledge is limited regarding whether nano-enabled basic substances accumulate in soils or sediments, move through irrigation runoff or dust, or engage with beneficial or harmful soil microbiota [61,67,69]. The EU Observatory for Nanomaterials recently highlighted the absence of explicit nanoform provisions in Regulation 1107/2009 [17] and in the Fertilizing Products Regulation (EU) 2019/1009 [70], creating uncertainty about how environmental fate, persistence, and nano–bio interactions should be assessed [71]. Another concern is the bioaccumulation and trophic transfer [67,68]. Although nanoparticles may also be taken up by plants and organisms through active processes, the extent to which particle size and surface charge strongly affect absorption and internal distribution remains unstudied [61]. In the European Union, risk assessment responsibilities are distributed across several authorities, with the European Food Safety Authority (EFSA) evaluating active substances used in PPP. Although EFSA issued nano-specific guidance in 2021, it applies only to food and feed uses and does not address environmental fate, leaving a critical gap for nano-agrochemicals [71].

Interactions with other agrochemicals signify another gap. Biostimulants, fertilizers, organic supplements, and pesticide residues, are commonly found in agricultural soils. The toxicity, synergy, or antagonistic effects of nano-enabled basic substances have not, however, been assessed. Considering the known ability of nanoparticles to carry, bind, or mobilize organic and inorganic pollutants, mixture effects may be considerable [67,68]. While some nanopesticides primarily function as biodegradable carriers and can be evaluated with traditional risk-assessment methodologies, higher co-formulant levels and modified release profiles are expected to raise exposure concerns [61].

From a human safety viewpoint, Singh et al. [67], Tran et al. [68], and Forest et al. [71], state that occupational exposure continues to be an overlooked issue. Many studies evaluate environmental toxicity or plant response; however, data on inhalation or dermal exposure for farm workers applying nanoformulations and for personnel involved in their production are lacking.

Regulatory frameworks add additional complexity. Currently, in the EU, nanoformulations are under the general definition of “substances” within REACH (EC 1907/2006) [72]. This term is further defined by the harmonized definition in Recommendation 2022/C 229/01/EU [20]. Regulation (EU) 2018/1881 [73], which modified REACH to require thorough characterization (size, shape, physicochemical properties), customized registration information, and specific chemical risk assessment (bioaccumulation, toxicity, environmental fate), and brought specific legal requirements for nanoforms into effect. The Biocidal Products Regulation (Regulation (EU) 528/2012) [74] and Recommendation 2022/692/EU [75], which improved the definition of nanomaterial, are in line with this framework [76].

Finally, there are gaps in socio-economic and agronomic validation. Despite frequent laboratory successes, very few natural-compound nanoparticle systems have been evaluated through field trials, robust economic cost–benefit analyses, or holistic life-cycle assessments [77,78,79]. Another significant challenge is scalability: transitioning from laboratory synthesis to industrial-scale, cost-effective, reproducible production of nano-enabled formulations requires technological advances in process engineering, quality control, and raw material stability [61,80]. Likewise, the lack of reliable data regarding farmer adoption drivers, market incentives, and compatibility with existing Integrated Pest Management (IPM) frameworks further limits real-world uptake [81].

## 4. Future Opportunities and Directions

The integration of basic substances and nano-enabled formulations requires coordinated development across the research, regulation, and practice levels. Policy and risk assessment frameworks are continuously evolving; hence, future innovation is needed to deliver field-relevant outcomes related to efficacy, safety, and sustainability [82,83,84]. With increasing demand for sustainable nanoparticle synthesis approaches by academia and industry sectors, there is an ever-growing interest in novel technologies that reduce environmental impact with concomitant gains in production efficiency. The identification and assessment of understudied substances, such as food-grade and pharmaceutical molecules, for antifungal and elicitor activities represents a priority among the research goals.

Central to this innovation is formulation science. Nanoencapsulation, solid lipid nanoparticles, and biopolymer coatings are examples of advances that improve the stability and delivery of active compounds under field conditions [82,83,85]. Such technologies can increase the persistence of active substances, enhance adhesion to plant surfaces, and provide controlled release, all of which improve the efficacy under variable climatic conditions. For example, NCS have shown improved antifungal activity and persistence compared with their bulk form. Similarly, chitosan nanoparticles and lipid-based nanocarriers have demonstrated high biocompatibility and low toxicity [84,86]. Such nanocarriers may allow for precision targeting of pathogens within IPM frameworks and contribute to reduced chemical inputs and lower environmental risk [87].

Future research should relate formulation design to plant–pathogen–environment interactions, focusing on how nanoformulations interact with leaf, fruit, and root, and how deposition, uptake, and persistence vary under different climatic and agronomic conditions [80,88]. This might be combined with molecular breeding and genome-editing technologies like Clustered Regularly Interspaced Short Palindromic Repeats-associated protein 9 (CRISPR-Cas9) as potential ways of making crops more responsive to nanocarrier-delivered inducers. Manipulation of key regulators of plant immunity, for example, Non-expressor of Pathogenesis-Related genes 1 (NPR1), WRKYGQK motif-containing transcription factor (WRKY), or Mitogen-Activated Protein Kinase (MAPK) components, may expand duration and strength of nanocarrier-delivered basic substances like chitosan [88,89,90].

To ensure that novel nanoformulations do not pose unanticipated environmental problems, adhesion to the Safe- and Sustainable-by-Design (SSbD) framework of the European Commission is crucial [91]. Comprehensive life cycle assessments and environmental fate studies should be used to quantify the sustainability benefits of nano-enabled technologies associated with conventional fungicides, considering effects on microbial diversity, soil health, and resource efficiency. The interactions between beneficial soil or phyllosphere microorganisms and basic substances made available by nanotechnology should receive particular attention in order to ensure that enhanced disease suppression does not endanger ecological equilibrium. A method that can be used for high-resolution studies of the structure of bio-macromolecules and their complexes, such as NPs, is cryo-electron microscopy. The study of dynamic biological processes at the nanoscale is made possible by the rapid freezing of materials in amorphous ice, which preserves their original structures. Thus, this method holds great promise for clarifying how NPs interact with biosystems and offer insights into their possible effects on environmental and human health [67]. To connect laboratory results with agronomic performance, large-scale validation is still necessary. Initiatives such as Agroecology Living Labs may provide participatory platforms for assessing nanoformulations under practical farming conditions. Simultaneously, digital agriculture and AI-based decision-support tools can optimize the timing, dosage, and application efficiency of nanoformulations and bioformulations to minimize off-target exposure and improve their cost-effectiveness [87,92,93].

The final adoption will be determined by socioeconomic viability. Production costs, lack of standardized regulations and a comprehensive safety framework, and the compatibility with the organic and low-input farming systems all have an impact on the commercial potential for natural compounds made available by nanotechnology. Policy initiatives under the Common Agricultural Policy can promote adoption and integration of sustainable agricultural practices through eco-schemes, collaborative partnerships, and sustainability labeling. As a result, public opinion and open communication are quite important. Although nanotechnology offers potent new solutions for long-term plant preservation, public trust depends on candid discussions regarding environmental impact, safety, and effectiveness [77,79]. In line with the EU’s SSbD agenda, international coordination through the Organization for Economic Co-operation and Development, and the Food and Agriculture Organization of the United Nations, as well as the United Nations’ Environment Program will be required to reach a consensus on standards, data requirements, and harmonization of risk assessment methodologies, according to Gottardo et al. [94] and Rauscher et al. [95]. Predictive modeling and cheminformatics are two technologies that could help with candidate selection. Digital risk databases and open-data infrastructures, supported by Horizon Europe’s Cluster 6, will allow better traceability and cross-border collaboration with harmonized platforms to collect, curate, and share nano-enabled agricultural input data throughout the EU. In this regard, allowing nano-enabled basic chemicals to contribute significantly to resilient and climate-adapted agroecosystems would depend on open risk communication, open data sharing, and early stakeholder involvement.

In the future, natural-compound nanoparticles have the potential to significantly broaden the scope of sustainable plant protection beyond current paradigms. Herein, the engineering of sophisticated nano–bio interfaces supporting programmable interactions with plant tissue and pathogens enables, for example, tissue-specific targeting, enhanced adhesion to leaves, and on-demand release triggered by specific environmental or pathogen-derived cues. The integration of NPs with phytohormones, microbial biocontrol agents, and elicitor molecules offers novel possibilities for synergistically boosting plant immunity without increasing chemical inputs [59,60,96]. High-throughput microfluidics, continuous-flow reactors, and modular biopolymer synthesis will be essential for manufacturing to render the natural-compound NP production process reproducible, scalable, and green, since this would yield a product with less batch variability while improving cost efficiency [97]. Computational tools ranging from cheminformatics and structure-activity modeling to multi-omics and machine learning will speed the discovery of novel natural compounds, optimize formulations, and predict nano-pathogen interaction before field deployment. At the systems scale, natural-compound nanoparticles are about to become key enablers of precision crop protection. Integrated with AI-based decision tools, sensor networks, and autonomous spraying technologies, NPs could dramatically lower inputs and improve spatiotemporal accuracy of application. Their intrinsic biodegradability and compliance with SSbD principles make them prime candidates for climate-smart and biodiversity-friendly agriculture [87,92,98,99].

## 5. Conclusions

The global commitment to reduce pesticide use becomes more widely adopted as society shifts toward reduced reliance on chemical inputs in food production. Practical, scalable, and ecologically friendly plant protection strategies, particularly those based on natural compounds, are required in order to meet sustainability and productivity targets. Although their existing forms frequently lack the persistence, stability, and delivery capability necessary for modern agriculture, basic substances offer one option to lessen reliance on synthetic fungicides. The limitation lies not in the chemistry of these substances but in how they are delivered. Consequently, innovation in delivery systems is expected to surpass the discovery of new active ingredients in importance, leading to a shift from an “*active-ingredient paradigm*” to “*active-delivery paradigm*” in sustainable crop protection. This is facilitated by nanotechnology and, in particular, green biodegradable and biocompatible nanocarriers. Nanoformulations enable more reliable solutions in IPM systems by enhancing stability, efficacy, and delivery of basic substances. Their adoption is challenging due to inconsistent production standards, lingering public concerns, and regulatory frameworks tailored to bulk chemicals. Their variable effectiveness against fungal plant pathogens showcases the need for focused research, optimization of delivery systems, and regulatory harmonization. This is the era of changing public perception that nanoformulations of natural compounds should be considered as straightforward substitutes to conventional pesticides. Adequate funding, cross-sector collaboration, and well-considered regulatory frameworks are prerequisites for basic substance-based nanoformulations to become prominent examples of agricultural innovation that balance production needs and environmental stewardship in the decade of sustainability action.

## Data Availability

Data are contained within the article.

## Supplementary Materials

The following supporting information can be downloaded at: https://www.mdpi.com/article/10.3390/plants15050689/s1, Table S1: List of approved uses of basic substances for fungal disease management in plants, under Regulation (EC) No. 1107/2009.

## Abbreviations

The following abbreviations are used in this manuscript:

EU	European Union
PPP	Plant Protection Products
REACH	Registration, Evaluation, Authorisation and Restriction of Chemicals
CLP	Classification, Labeling and Packaging of Substances and Mixtures
NPs	Nanoparticles
ROS	Reactive oxygen species
SA	Salicylic acid
JA	Jasmonic acid
SAR	Systemic acquired resistance
ISR	Induced systemic resistance
NCS	Chitosan nanoparticles
EFSA	European Food Safety Authority
IPM	Integrated Pest Management
CRISPR/Cas9	Clustered Regularly Interspaced Short Palindromic Repeats-associated protein 9
NPR1	Non-expressor of Pathogenesis-Related genes 1
WRKY	WRKYGQK motif-containing transcription factor
MAPK	Mitogen-Activated Protein Kinase
SSbD	Safe- and Sustainable-by-Design
